# Artificial placenta support of extremely preterm ovine fetuses at the border of viability for up to 336 hours with maintenance of systemic circulation but reduced somatic and organ growth

**DOI:** 10.3389/fphys.2023.1219185

**Published:** 2023-08-24

**Authors:** Haruo Usuda, Hideyuki Ikeda, Shimpei Watanabe, Shinichi Sato, Erin L. Fee, Sean W. D. Carter, Yusaku Kumagai, Yuya Saito, Tsukasa Takahashi, Yuki Takahashi, Shinichi Kawamura, Takushi Hanita, Masatoshi Saito, Atsuo Kikuchi, Mahesh A. Choolani, Nobuo Yaegashi, Matthew W. Kemp

**Affiliations:** ^1^ Division of Obstetrics and Gynecology, The University of Western Australia, Crawley, WA, Australia; ^2^ Center for Perinatal and Neonatal Medicine, Tohoku University Hospital, Sendai, Miyagi, Japan; ^3^ Department of Obstetrics and Gynaecology, Yong Loo Lin School of Medicine, National University of Singapore, Singapore, Singapore; ^4^ Nipro Corporation, Osaka, Japan; ^5^ School of Veterinary and Life Sciences, Murdoch University, Perth, WA, Australia

**Keywords:** artificial placenta, extremely preterm infant, fetal growth, extracorporeal membranous oxygenator, fetal circulation, growth restriction, sheep model, preterm birth

## Abstract

**Introduction:** Artificial placenta therapy (APT) is an experimental life support system to improve outcomes for extremely preterm infants (EPI) less than 1,000 g by obviating the need for pulmonary gas exchange. There are presently no long-term survival data for EPI supported with APT. To address this, we aimed to maintain 95d-GA (GA; term-150d) sheep fetuses for up to 2 weeks using our APT system.

**Methods:** Pregnant ewes (*n* = 6) carrying singleton fetuses underwent surgical delivery at 95d GA. Fetuses were adapted to APT and maintained for up to 2 weeks with constant monitoring of key physiological parameters and extensive time-course blood and urine sampling, and ultrasound assessments. Six age-matched *in-utero* fetuses served as controls. Data were tested for group differences with ANOVA.

**Results:** Six APT Group fetuses (100%) were adapted to APT successfully. The mean BW at the initiation of APT was 656 ± 42 g. Mean survival was 250 ± 72 h (Max 336 h) with systemic circulation and key physiological parameters maintained mostly within normal ranges. APT fetuses had active movements and urine output constantly exceeded infusion volume over the experiment. At delivery, there were no differences in BW (with edema in three APT group animals), brain weight, or femur length between APT and *in-utero* Control animals. Organ weights and humerus lengths were significantly reduced in the APT group (*p* < 0.05). Albumin, IGF-1, and phosphorus were significantly decreased in the APT group (*p* < 0.05). No cases of positive blood culture were detected.

**Conclusion:** We report the longest use of APT to maintain extremely preterm fetuses to date. Fetal systemic circulation was maintained without infection, but growth was abnormal. This achievement suggests a need to focus not only on cardiovascular stability and health but also on the optimization of fetal growth and organ development. This new challenge will need to be overcome prior to the clinical translation of this technology.

## Introduction

Around 15 million babies (a global preterm birth rate of about 11%) are born preterm each year (Howson et al., 2013). The comparably small percentage of infants born at the present border of viability (21–24 weeks gestation) are at significant risk of death or life-compromising complications (Stoll et al., 2015; Zeitlin et al., 2016; Glass et al., 2015; Bell et al., 2022). For extremely preterm infants (EPIs), their underdeveloped cardiopulmonary system likely causes higher rates of death and disease in the precocious transition to pulmonary respiration. Development of life support technology that does not force the transition to pulmonary gas exchange for EPIs, contingent upon rapid adjustment of the cardiovascular system, may allow improved outcomes for EPIs (Stoll et al., 2010; Glass et al., 2015).

With this objective in mind, we have worked to develop an experimental treatment platform for EPIs, artificial placenta therapy (APT). The nucleus of this concept is to treat EPIs as fetuses, rather than as small babies, and to avoid the use of pulmonary gas exchange. In previous reports based on our APT system, we demonstrated healthy fetal survival in mature sheep fetuses (115 d, 2000 g) for a period of 168 h, and further extended that to 336 h in a subsequent set of unpublished experiments (Miura et al., 2015; Miura et al., 2016; Miura et al., 2017; Usuda et al., 2017). Using a pump-based system, Kuwabara and Unno previously reported long-term survival in mature goat fetuses with long-term transition to mechanical ventilation (Unno et al., 1993). Similarly, Partridge *et al.* also reported successful maintenance of mature fetuses (116 d, 2300 g) using a fetal heart-driven platform similar to our own for up to 28 days before transition to pulmonary ventilation (Partridge et al., 2017).

Despite these important achievements, most studies have employed fetal sheep or goats significantly larger and more mature than EPIs, the likely clinical candidates for APT. Recently, Charest-Pekeski *et al.* used late preterm fetal pigs with a pumpless APT system (98 d/115d-term: 743 ± 350 g: 11 ± 13 h survival) and a pump-driven APT system (102 d: 616 ± 139 g: 46.4 ± 46.8 h survival); they reported fetal death due to progressive decline in the umbilical vein (UV) flow and oxygen delivery (Charest-Pekeski et al., 2021; Charest-Pekeski et al., 2022). Hornick *et al.* also demonstrated the feasibility of catheterizing the extremely preterm lamb umbilical cord, and maintained fetuses on a pumpless APT system driven by fetal cardiac heart (85-96d/145 d-term: 641 ± 71 g: 140 ± 7 h survival); their report highlighted key challenges in relation to fetal death with progressive hydrops on and after around 72 h, presumably due to cardiac failure (Hornick et al., 2019). A series of pre-determined 5-day studies using healthy and compromised 95d fetuses weighing approximately 600 g were also performed using our APT platform (Usuda et al., 2019; Usuda et al., 2020). Cardiac performance, blood gas parameters, and gross somatic growth in AP-treated fetuses were all found to be equivalent to that of age-matched, *in-utero* controls delivered at 100 days’ gestation. Fetuses were not hydropic. Infection and overt brain injury were absent (Usuda et al., 2019). Although short-term healthy survival has been demonstrated with our APT system, data informing the longer duration survival of the EPI with artificial placenta technology is not available (Usuda et al., 2022a).

Therefore, we aimed to assess the ability of our APT platform to support clinically relevant, extremely preterm lambs at 93–94 days gestational age (dGA, ∼24 weeks GA human equivalent in terms of weight and size), weighing approximately 600 g for a period of up to 336 h. The following primary endpoints were chosen to evaluate platform utility: i) maintenance of key physiological parameters and systemic circulation; ii) absence of infection; iii) growth patterns matching that of age-matched *in-utero* control fetuses.

## Material and methods

### Experimental protocol

#### APT group

Merino-cross ewes with timed, singleton pregnancies (n = 6) were surgically delivered at 93 or 94 dGA following ultrasound assessment.

#### Intrauterine control group

Age-matched, *in-utero* singleton fetuses served as controls (n = 8). After a final ultrasound measurement, control animals were delivered and euthanized with an intravenous bolus of pentobarbitone (160 mg/kg) to allow comparative measurement of body and organ weights. Fetal blood and plasma were collected at delivery to perform blood counts, (including differential leukocyte count), and biochemical and endocrine analyses.

#### Acquisition of blood gas reference data

Reliable fetal blood gas data could not be obtained from *in-utero* Control animals above due to the euthanasia of ewe and fetus before delivery. Therefore, the intra-uterine control blood gas data presented herein (Table 2) were obtained as a reference set from null-treatment fetuses previously collected as part of our ovine databank (Usuda et al., 2019). Briefly, thirteen merino-cross ewes with timed, singleton pregnancies (97 ± 2 d GA) were premedicated, anesthetized, intubated, and ventilated (1%–2% isoflurane in 21% oxygen inhaled, tidal volume 10 mL/kg 8–10 breaths/minute) for at least 30 min. Then, after a maternal laparotomy and hysterotomy, a fetal blood sample was taken from an umbilical artery for blood gas analysis.

### Surgical delivery

Ewes were fasted for 12 h before surgery with *ad libitum* access to water. Ewes were premedicated, anesthetized, intubated, and ventilated (acepromazine 0.03 mg/kg and buprenorphine 0.01 mg/kg intramuscularly, midazolam 0.25 mg/kg and ketamine 5 mg/kg intravenously, 1%–2% isoflurane in 100% oxygen inhaled, tidal volume 10 ml/kg 8–10 breaths/minute) during the surgical procedure. Intravenous fluids (0.9% NaCl) were administered at a rate of 10 mL/kg/h. The ewe’s abdomen was clipped to expose the skin and thoroughly prepared for surgery as described previously (Usuda et al., 2019; Usuda et al., 2020). After a maternal laparotomy and hysterotomy, the fetuses were placed inside a sterilized artificial uterine bag (Nipro Corporation, Osaka, Japan), with care taken to ensure umbilical cord patency. Fetuses were intermittently bathed with sterile saline warmed to 40°C. The catheterization procedure was performed prior to delivery as follows, in a procedure taking approximately 10–15 min: i) one umbilical artery was cannulated with a 10 Fr custom-made arterial catheter (Nipro Corporation, Osaka, Japan) and secured approximately 7–8 cm outside the umbilical ring. The tip of the arterial catheter was sited approximately 5–6 cm from the umbilical ring; ii) one umbilical vein was then quickly cannulated with a 10 Fr custom-made venous catheter (Nipro Corporation, Osaka, Japan) and secured approximately 1.5–2 cm external to the umbilical ring. The tip of venous catheter was sited approximately 1–1.5 cm past the umbilical ring. The fetuses were then attached to one membranous oxygenator; iii) a second umbilical artery was catheterized and then connected to the circuit; iv) the urachus was cannulated to the bladder with a 3 or 4Fr polyvinyl catheter (Atom medical corporation, Tokyo, Japan) and: v) lastly, the fetus was transferred to the maintenance platform. The bag was closed and promptly filled with synthetic AF. Ewes in both the APT and *in-utero* Control Groups were euthanized with an intravenous bolus of pentobarbitone (160 mg/kg).

### APT system components

1) Artificial placenta

The artificial placenta circuit was composed of three main parts: i) two outflow 45 cm tubes (1/4 inch); ii) one custom-made membranous oxygenator made of polyolefin hollow fiber; and iii) a 50 cm inflow tube (1/4 inch) (all Nipro Corporation, Osaka, Japan). Polyvinyl chloride tubes coated with a synthetic polymer were used for both the inflow and outflow tubes. The circuit was primed with 70 ml of heparinized maternal blood. The calculated membrane surface area for gas exchange was 0.21 m^2^. The system was driven by the fetal heart and external pumps were not used to maintain or support the circuit flow.

2) Amniotic Fluid

Sterile synthetic amniotic fluid (AF) was aseptically prepared as follows: pH: 7.19 ± 0.06, Na+: 115 ± 3 mEq/L, Cl-: 109 ± 3 mEq/L, K+: 5.6 ± 0.1 mEq/L, Ca2+: 0.44 ± 0.03 mEq/L, all values for pH and electrolytes represent group mean ± standard deviation (SD). The AF was preheated to 39.5°C–40.0°C and UV-treated. The AF bath was filled with 6 L of synthetic AF and warmed constantly by two heaters. Heaters were installed at the top (radiant warmer) and at the bottom (contact heat pad) of the AF bath. After the fetus was submerged, AF was maintained at a constant temperature of 38.5°C ± 0.5°C (group mean ± SD). The AF bath was rinsed and AF was replaced every 8 h after the start of the APT with 30 L of new synthetic AF which had been UV-treated and micro-filtered (0.2 μm).

3) Intravenous nutrition

Intravenous nutrition consisted of glucose (10%–11%), amino acids (1.8–3.6 g/day, estimated within 2.5–3 g/kg/d, Amizet B; Terumo Corporation, Tokyo, Japan), lipid (0.1g/day, intralipid 20%; Fresenius Kabi Australia, Sydney, Australia), vitamin compounds (1/6 vial/day, Daimedin multi inj; Nichi-Iko Pharmaceutical, Tokyo, Japan), micronutrients (0.1 mL/d, Cizanarine N Inj; Nissin Pharmaceutical, Yamagata, Japan), Calcium (Ca; 45–83 mg/d, estimated within 65–100 mg/kg/day), Phosphorus (P; 35–60 mg/day, estimated within 50–80 mg/kg/d) and magnesium (Mg: 6–12 mg/d, estimated within 5–10 mg/kg/d) to provide 48–75 kcal/d (estimated around 65–75 kcal/kg/d) described as previously (Boullata et al., 2014; Thomas, 2016; Usuda et al., 2019). Glucose (Glu) levels were maintained between 1 and 7 mmol/L to prevent hypo and hyperglycemia (Ramel and Rao, 2020). Blood urea nitrogen (BUN) levels were managed between 9 and 40 mg/Dl (Trends in blood urea nitrogen, 2017; Mathes et al., 2018; Giretti et al., 2021). Average infusion volumes (ml/h) for each APT animal were calculated by the formula: sum of infusion (ml)/experimental duration (h). The average amino acid administration (g/day) was calculated by the formula: sum of amino acids administration (g)/experimental duration (h) x 24.

### Collection of maternal blood for fetal transfusion

Meropenem (1 g/dose) was administered to each ewe following the induction of anesthesia. 100 ml of venous blood (approximately 2%–3% of total circulating blood volume for ewes) was aseptically collected from the jugular vein prior to surgery commencing. Whole blood was immediately heparinized (10 U/ml) and then used for priming of the artificial placenta circuit (Rasmusen, 1962). A further 400 ml of whole blood was collected after fetal delivery using a triple-bag blood transfusion system (T331150, Fresenius Kabi, Mount Kuring-gai, Australia). Packed red cells (RBC) were preserved at 4°C prior to use. Fresh plasma was frozen (FFP) at −80°C and defrosted on demand. RBC transfusions generated with maternal blood were performed (10 ml/kg/dose) when hemoglobin values fell below 9 g/dl. FFP was administered to compensate for bleeding as necessary.

### Maintenance after delivery

APT Group fetuses were maintained and observed in parallel by a single investigator on a rotating 12 h shift. 24 h after commencement of APT, normal intermittent active fetal swallowing movements, breathing movements, gross fetal body movements, and flexure and extension of limbs were assessed as indicators of biophysical condition at least once every 6 h. The presence of edema or bleeding was determined by ultrasound, and by gross examination during necropsy after the experiment concluded. Lambs were continuously treated with intravenous argatroban (0.2–0.7 μg/kg/h) to prevent blood coagulation, with the dose adjusted after stabilization in an attempt to maintain an activated partial thromboplastin time (APTT) of 100–150 s. Oxygen supply to the membranous oxygenators was adjusted to maintain fetal PaO2 between 20 and 35 mmHg (Comline and Silver, 1970). Hydrocortisone (3 mg) was intravenously administered to the fetuses immediately after the induction of the APT, followed by daily administrations at a dose of 5.4 mg/day (estimated 8–9 mg/kg/day, ∼24 h), 3.0 mg (estimated 4–5 mg/kg/d, ∼72 h) and 2.1 mg (1.5–3 mg/kg/d) to manage fetal critical refractory hypotension as described previously (Usuda et al., 2019; Usuda et al., 2020). Prostaglandin E_1_ (PGE_1_) (40 ng/kg/min, Tandetron; Takata Pharmaceutical, Saitama, Japan) was continuously administered after delivery. Midazolam was continuously administered for the first 6 h (0.15 mg/kg/h), or if bleeding was suspected, to sedate the fetus.

An APT circuit clamp was used to adjust circuit blood flow and aimed to keep it below 350 ml/kg/min (based on fetal weights at the commencement of the APT). The clamp was released when more than a 15% decrease in circuit blood flow within 6 h was observed. A circuit flow of less than 150 ml/min (regardless of cause) resulted in the intravenous administration of higher-dose nitroglycerin (2–10 μg/kg/min, Millisrol Inj. Nippon Kayaku Co., Ltd., Tokyo, Japan) so as to prevent constriction of umbilical vessels and maintain circuit blood flow (Usuda et al., 2022b).

### Scheduling of euthanasia

Fetuses were maintained with APT for up to 336 h, followed by euthanasia with intravenous pentobarbitone (160 mg/kg/dose) for measurements of body and organ weight (brain, heart, lung, liver, and kidney) and tissue sample collection. When both APT circuit blood flow was less than 150 ml/min and fetal lactate level was more than 4 mmol/l (or estimated to exceed that value within the next 6 h), fetuses were immediately euthanized for analysis.

### Prevention of infection

Bacterial infection was determined based on a positive finding of bacteremia by microbial cultures. To prevent infection, meropenem (10–15 mg/dose, Ranbaxy; Sydney, Australia) was administered intravenously to the fetuses every 6 h. Intravenous fluconazole (4 mg/kg/dose, Fluconazole-Claris; AFT Pharmaceuticals Pty Ltd., Sydney, Australia) was administered to the fetuses every 24 h.

### Physiological, hematological, biochemical, and microbiological data acquisition

Fetal heart rate (HR: beats/min) and mean arterial circuit blood pressure (mBP: mmHg) were continuously monitored and recorded using a SurgiVet monitor (Smiths Medical, St. Paul, MN). Circuit blood flow (ml/min) was continuously monitored using electromagnetic flow sensors (Transonic 400-Series, Transonic Systems Inc., Ithaca, NY) attached to the arterial positions of the blood circuit, and recorded using a Power-Lab (ADInstruments, Dunedin, New Zealand). Fetal umbilical arterial blood gasses: pH, base excess (BE), pCO_2_, pO_2_, O_2_ saturation (SO_2_), O_2_ content (CtO_2_), hemoglobin (Hb), sodium ion (Na^+^), potassium ion (K^+^), calcium ion (Ca^2+^), chloride ion (Cl^−^), lactate (Lac), Glu level (Siemens RapidPoint 500, Munich, Germany) and APTT (Hemochron Jr., Accriva Diagnostics, San Diego, CA) were measured at least every 6 h. Fetal umbilical arterial blood samples were collected every 24 h following the induction of the APT.

Hematological analyses performed included: white blood cell counts (WBC), and differential leukocyte counts; biochemical analyses performed included: aspartate aminotransferase (AST), alanine aminotransferase (ALT), gamma-glutamyl transpeptidase (GGTP), glutamate dehydrogenase (GLDH), blood urea nitrogen (BUN), creatinine (Cre), BUN/creatinine ratio (BUN/Cre), total bilirubin (T-bil), albumin (Alb), alkaline phosphatase (ALP), Ca, P, Mg; endocrine analyses performed included: cortisol, adrenocorticotropic hormone (ACTH), insulin-like growth factor 1 (IGF-1); and microbiological analyses performed included: anaerobic and aerobic cultures. Studies were performed by an independent clinical pathology laboratory (Vetpath, Perth, Australia). To prevent hypoxia due to anemia, all sampling was made volume-neutral via the addition of packed red cells and fresh-frozen plasma.

### Urine data acquisition

The urachal catheter was connected to a collection bag. Voiding volume was measured and urine samples were collected every 24 h. The average voiding volume (ml/h) was calculated by the following formula: sum of daily voiding volume (ml)/experimental duration (h). Concentrations of Cre and P in urine samples were measured by an independent clinical pathology laboratory (Vetpath, Perth, Australia). Tubular reabsorption of phosphorus (TRP) was calculated using the formula [1-(urine P/serum P)/(urine Cre/serum Cre)] x 100 (%) (Acar et al., 2015). Protein concentration in urine samples was quantified with a Qubit 2.0 fluorometer (Life Technologies) using a protein assay kit (Life Technologies). Average protein excretion into urine (g/d) was calculated by the formula: sum of daily voiding volume (ml) x protein concentration (g/ml)/experimental duration (h) × 24.

### Ultrasound assessment

Ultrasound assessments were performed regularly for cardiac function (d0, 7, then each day and d14), long bone length (d0, 7, then each day and d14), and edema (daily) in addition to emergent assessments when bleeding was suspected. Measurements were conducted with a Philips CX50 system, S5-1 phased array probe (both Philips Healthcare, Netherlands), and associated obstetrics software.

#### Cardiac function

For APT Group animal measurements, the ultrasound beam was focused through the transparent AF bath. For Control Group animal measurements, ewes were held in a dorsal recumbency and the fetal position from the ventral aspect was confirmed. The ultrasound beam was focused to obtain a basal four-chamber view, five-chamber view, left ventricular outflow tract (LVOT), right ventricular outflow tract (RVOT) view, or three-vessel (3 V) view to check the following items described previously (Usuda et al., 2019; Usuda et al., 2020).

Briefly, the distance between the attachment point of the mitral valve on the epicardium to the attachment point of the tricuspid valve on the epicardium was measured in a four-chamber view as total cardiac dimension (TCD). Trans tricuspid and trans mitral inflow were measured using Doppler echocardiography to assess the peak early diastolic filling (E wave) and late diastolic filling (A wave) velocities in the calculation of each E/A ratio. Cardiac time intervals including isovolumetric contraction time (ICT), ejection time (ET), and isovolumetric relaxation time (IRT) were measured for the left ventricle. Myocardial performance index (MPI) was calculated using the formula defined as MPI = ICT + IRT/ET.

Pulsed Doppler tracings were obtained at the point of the inferior *vena cava* orifice entering the right atrium. Peak velocity during atrial contraction (A), which frequently has reversed blood velocities away from the heart, and peak velocity during ventricular systole (S) were measured from the recorded flow velocity waveform, and the A/S ratio was calculated to obtain the preload index (PLI) (Kanzaki and Chiba, 1990; Gudmundsson, 1999; Kanagawa et al., 2002; Hidaka et al., 2009). The internal diameter of *ductus arteriosus* was measured at the confluence of the descending aorta. Color flow Doppler imaging was used to detect the blood flow direction through the *ductus arteriosus*. Blood flow from the pulmonary artery to the descending aorta was determined as right-to-left directional flow.

#### Long-bone length and edema

The ultrasound beam was focused parallel to the major axis of the humerus and the femur. Shortly after euthanasia, final physical measurements of the humerus and femur were performed and used as end-of-experiment data. Long-bone length gains were calculated using the following formula: bone length (conclusion)–bone length (d0).

Edema was defined as follows: skin thickness greater than 5 mm as positive (+) (Snowise et al., 2018).

### Laboratory analyses

#### Enzyme-linked immunosorbent assays

Parathyroid hormone (PTH) concentrations were measured using fetal plasma. Commercial kits from MyBioSource (San Diego, US), with washing performed on a Biosan plate washer (3D-IW8, Inteliwasher, Biosan, Riga, Latvia) were used as previously described (Usuda et al., 2017). Standards (calibration curve *R* ^2^ > 0.97) were assayed in triplicate (average coefficient of variation 6.1%) and samples were assayed in duplicate. The sensitivity in the assay was 1 pg/ml. Assays were performed in accordance with the manufacturer’s instructions, with absorbance at 450 nm read on a HiPo MPP-96 microplate photometer (Biosan, Riga, Latvia).

### Statistical analyses

All values are expressed as either mean ± one standard deviation or median [IQR]. Statistical analyses were performed using IBM SPSS for Windows, Version 23.0 (IBM Corporation, Armonk, NY). A Chi-Square test was used to test the differences in nominal values between the two groups. All numerical data were tested for normality with Shapiro-Wilk tests. In the comparison of two groups, between-group differences in parametric data were tested for significance with *t*-tests, while Mann-Whitney U tests were used for non-parametric data. Significance was accepted at *p* < 0.05. A 95% confidence interval was shown with *p*-value for numerical data.

## Results

### Physiological variables

**FIGURE 1:**
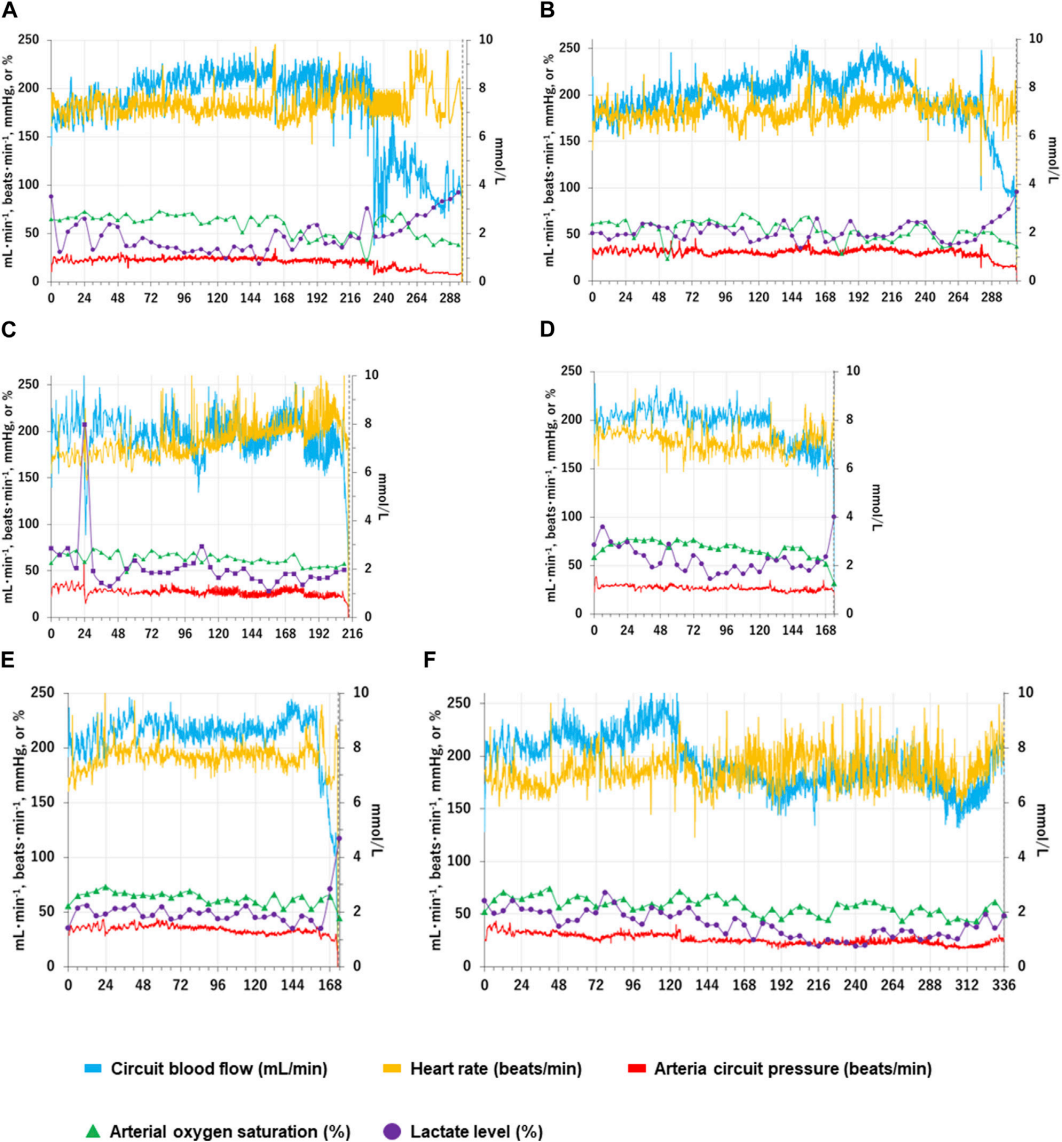
Changes in individual fetal physiological and biochemical variables over time in the APT group. Fetal physiological and biochemical variables over time for each APT animal (Cases A-F) are shown individually as Panels **(A–F)**. The horizontal axis represents the time after the induction of APT (h). The blue solid lines show total oxygenator (circuit) blood flow (ml/min); the yellow solid lines show heart rate (beats/min); the red solid lines show mean arterial circuit pressure (mmHg); The green closed triangles show arterial oxygen saturation (%); the purple closed squares show blood lactate level (mmol/l). Blood lactate levels are shown on the right scale. The vertical black dotted lines indicate the time of euthanasia for individual APT animals.

**FIGURE 2:**
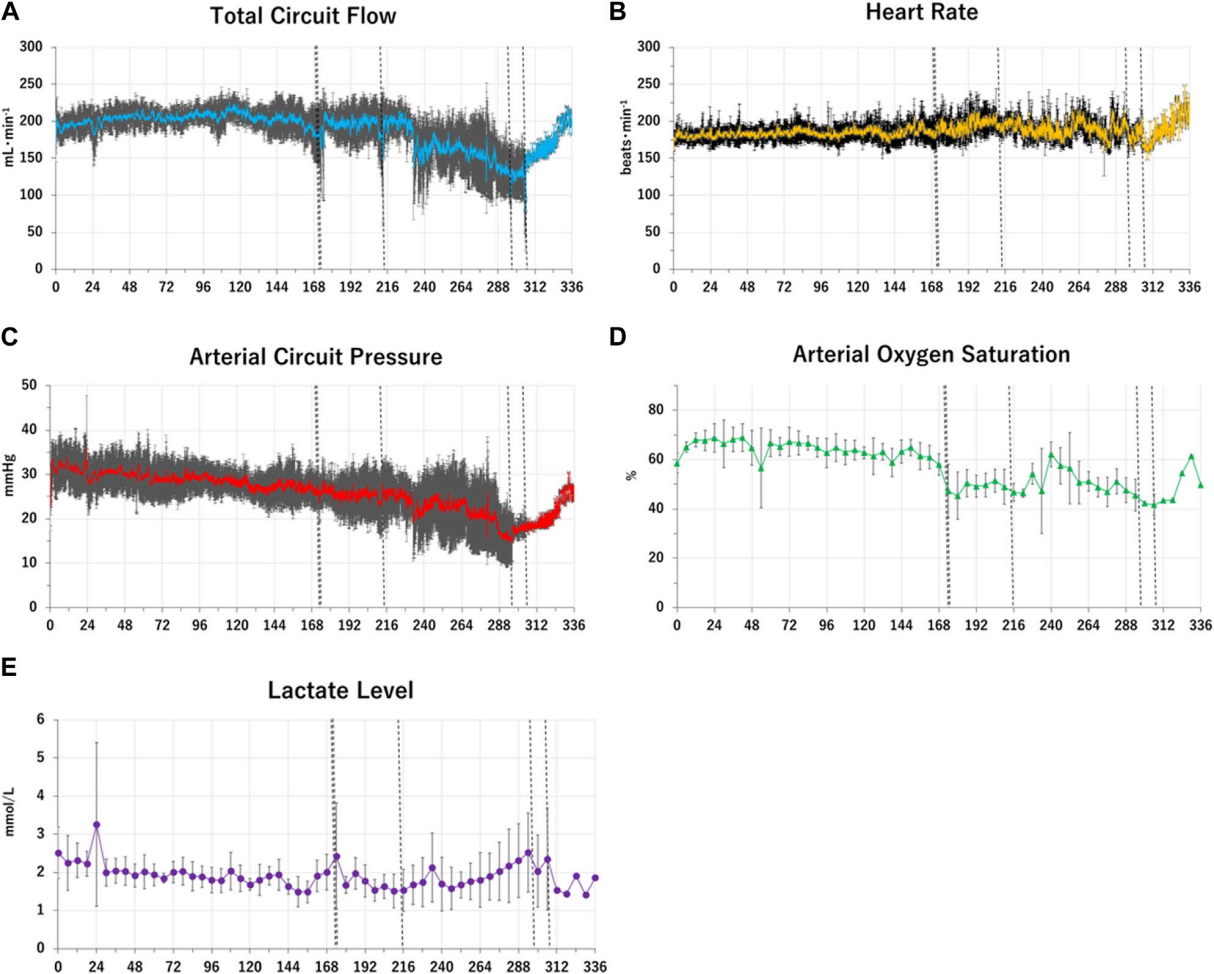
Changes in group means of fetal physiological and biochemical variables over time in the APT Group. Group mean values of key fetal physiological and biochemical variables over time in the APT animals (Case A-F) are shown as Panel **(A–E)**. The horizontal axis represents the time after the induction of APT (h). Panel **(A)**: the blue solid lines show total oxygenator (circuit) blood flow (ml/min). Panel **(B)**: the yellow solid lines show heart rate (beats/min). Panel **(C)**: the red solid lines show mean arterial circuit pressure (mmHg). Panel **(D)**: the green closed triangles show arterial oxygen saturation (%). Panel **(E)**: the purple closed squares show blood lactate level (mmol/l). Standard deviations of each variable at each time point were shown as black lines and whiskers. The vertical black dotted lines indicate the times at euthanasia for individual APT animal (173 h; animal case E, 174 h; animal case D, 213 h; animal case C, 296 h; animal case A, 306 h; animal case B). After euthanasia each animal ceased contributing toward the mean values.

Six APT Group lambs (100%) were adapted to APT successfully. The mean body weight at the commencement of APT was 656 ± 42 g. Mean survival was 250 ± 72 h (Table 1). One of six APT Group fetuses survived for the pre-determined 336 h experimental duration. Three of six fetuses were euthanized after acute or subacute bleeding from the bladder. A further two fetuses were euthanized due to umbilical artery vasoconstriction. All corresponding Control Group animals were euthanized at the same dGA as their paired APT Group fetuses. Individual and group mean of key physiological data are presented in Figures 1, 2.

**TABLE 1:** Comparison of fetal data at necropsy. Values are expressed as the group mean ± SD. *p* < 0.05 was considered a significant difference vs. value for the *in-utero* Control group. Long-bone length gains were calculated using the formula: bone length (conclusion)–bone length (d0).

	Control group	APT group	Statistical test	*p*-value [95% CI]
Number	8	6		
Gestational age at the induction of APT (d)	—	93.7 ± 0.5	—	—
Survival time (h)	—	250 ± 72	—	—
Gestational age at necropsy (d)	105.9 ± 2.4	104.5 ± 3.2	Mann-Whitney U	0.282
Sex (male/female)	3/4*	1/5	—	—
Body weight (Induction of APT) (g)	—	656 ± 42	—	—
Body weight (necropsy) (g)	1,356 ± 179	1,323 ± 116	t-test	0.702
Brain weight (g)	28.2 ± 2.6	29.0 ± 1.2	t-test	0.46
Heart weight (g)	8.8 ± 1.9	6.9 ± 1.5	t-test	0.063
Lung weight (g)	45.7 ± 6.6	37.0 ± 4.0*	t-test	0.015 [−15.3 to −2.0]
Liver weight (g)	75.25 ± 13.1	53.0 ± 4.5*	t-test	0.002 [-33.5 to −11.0]
Kidney weight (g)	12.9 ± 1.7	9.5 ± 2.2*	t-test	0.007 [−5.7 to −1.1]
Humerus length gain (cm)	4.8 ± 1.9	2.9 ± 1.0*	t-test	0.036 [−3.6 to −0.10]
Femur length gain (cm)	5.5 ± 2.6	3.8 ± 0.8	t-test	0.113

There were five female and one male fetuses in the APT Group. There were four female and three male fetuses in the Control Group. The sex of one Control Group fetus was not recorded. There were no significant differences in gestational age, fetal body weight, brain weight, or femur length gain at the end of the experiment between the APT Group and *in-utero* Control Group animals over the experimental period. Fetal lung weight, liver weight, kidney weight, and humerus length gain in the APT Group were less than those of the *in-utero* Control Group (Table 1). The mean difference in heart weight between groups was non-significant (*p* = 0.063).

Cord blood gas data; pH, pCO_2_, pO_2_, BE, SO_2_, CtO_2_, Hb, Na^+^, K^+^, Ca^2+^, Cl^−^, Lac, and Glu remained clinically acceptable and comparable to the normal reference data (Table 2). APTT was maintained mostly within target ranges.

**TABLE 2:** Blood gas data from APT and Reference Groups. Six APT Group animals were analyzed throughout the experiment. Umbilical arterial blood was collected for blood gas data every 6 h after the start of the APT. Values are expressed as the group mean ± SD. SO_2_, O_2_ saturation; CtO_2_, O_2_ content = hemoglobin (g/dl) × 1.34 × SpO_2_ (%)/100+ pO_2_ × 0.003; Na^+^, sodium ion; K^+^, potassium ion; Ca^2+^, calcium ion; Cl^−^, chloride ion; APTT, activated partial thromboplastin time. Reference data were obtained from thirteen age-matched (97 ± 2 dGA), null-treatment fetuses, which were previously collected for our ovine databank and not run concurrently alongside the APT group animals.

	References	APT group
Animal number	13	6
Range of gestational age in the collection of umbilical arterial blood (d)	95–99	93–108
pH	7.38 ± 0.03	7.42 ± 0.05
pCO_2_ (Torr)	42.4 ± 2.9	41.7 ± 4.0
pO_2_ (Torr)	25.1 ± 2.1	30.4 ± 4.2
Base excess (mmol/L)	‐0.2 ± 1.8	1.9 ± 3.4
SO_2_ (%)	65.5 ± 5.9	59.6 ± 10.0
Hemoglobin (g/L)	93 ± 9	107 ± 13
CtO_2_ (mmol/L)	8.9 ± 1.2	8.6 ± 2.1
Na^+^ (mmol/L)	134 ± 3.1	140 ± 6.4
K^+^ (mmol/L)	4.0 ± 0.3	3.8 ± 0.4
Ca^2+^ (mmol/L)	1.4 ± 0.1	1.3 ± 0.2
Cl^−^ (mmol/L)	103 ± 1.7	113 ± 4.1
Lactate (mmol/L)	1.7 ± 0.4	1.9 ± 0.7
Glucose (mmol/L)	0.8 ± 0.3	3.5 ± 1.6
APTT (sec)	-	120 ± 25

### Hematological, biochemical, and microbiological variables

There were no significant differences in WBC, neutrophil, lymphocyte, AST, ALT, GGTP, GLDH, T-bil, Cre, and ACTH levels between the APT Group and *in-utero* Control Group fetuses at the end of experiments. BUN, BUN/Cre ratio, PTH, and cortisol were significantly higher, while Alb, IGF-1, ALP, Ca, P, and Mg were significantly lower in the APT Group fetuses than those in the *in-utero* Control Group (Table 3). No bacteria were identified from blood or synthetic AF in the any of six AP Group animals at the end of the experiments (Table 4).

**TABLE 3:** Comparison of fetal hematological and biochemical variables at the end of experiment. Eight Control Group animals and Six APT Group animals were analyzed. Normally distributed values are expressed as the group mean ± SD, while non-parametric values are expressed as the group median [IQR]. Significant differences in values for the *in-utero* Control group are indicated: *, *p* < 0.05 and [95% confidence interval]. AST, aspartate aminotransferase; ALT, alanine aminotransferase, GGTP, gamma-glutamyl transpeptidase; GLDH, glutamate dehydrogenase; BUN, blood urea nitrogen; Cre, creatinine; IGF-1, insulin-like growth factor 1; ALP, Alkaline phosphatase; PTH, parathyroid hormone; ACTH, adrenocorticotropic hormone. Tubular reabsorption of phosphorus (TRP) was calculated using the following formula: 1-(urine phosphorus/serum phosphorus)/(urine creatinine/serum creatinine) x 100 (%).

	Control group	APT group	Statistical test	*p*-value
White blood cell counts (/μl)	1750 [1,375–2,200]	1,400 [1,400–1,475]	Mann-Whitney U	0.121
Neutrophil ratio (%)	21.1 ± 7.2	27.8 ± 14.4	t-test	0.37
Lymphocyte ratio (%)	75.0 ± 9.0	59.8 ± 15.7	t-test	0.091
AST (U/l)	26 ± 14	41 ± 25	t-test	0.108
ALT (U/l)	1 [1–2.3]	7.5 [2.5–17]	Mann-Whitney U	0.081
GGTP (U/l)	20 ± 8	57 ± 55	t-test	0.2
GLDH (U/l)	5 ± 3	10 ± 11	t-test	0.194
Total bilirubin (mg/dl)	0.47 [0.34–0.60]	0.23 [0.09–0.42]	Mann-Whitney U	0.181
BUN (mg/dl)	10.5 [10.3–13.4]	18.1 [16.7–21.1] *	Mann-Whitney U	0.02
Creatinine (mg/d/l)	1.1 ± 0.2	0.9 ± 0.5	t-test	0.372
BUN/Cre ratio	10.9 [9.5–12.4]	28.0 [26.3–30.5]*	Mann-Whitney U	0.029
Albumin (g/dl)	1.74 ± 0.1	1.2 ± 0.34*	t-test	0.014 [-0.92–0.15]
IGF-1 (μg/l)	140 ± 18	17 ± 7*	t-test	0.001 [-148–105]
ALP (U/l)	169 ± 24	102 ± 19*	t-test	0.001 [-104–43]
Calcium (mg/dl)	12.7 [12.6–12.9]	8.6 [7.7–9.2]*	Mann-Whitney U	0.001
Phosphorus (mg/dl)	7.8 ± 0.7	5.2 ± 0.6*	t-test	0.001 [-3.5–1.7]
Magnesium (mg/dl)	2.5 ± 0.2	2.1 ± 0.2*	t-test	0.020 [-0.6–0.1]
PTH (pg/ml)	35.9 [33.4–36.5]	46 [40.7–56.6]*	Mann-Whitney U	0.004
Tubular reabsorption of phosphorus (%)	-	96.1 ± 2.8	-	-
Cortisol (nmol/l)	15.3 ± 6.5	377.7 ± 133.3*	t-test	0.002 [210.3–516.7]
ACTH (pg/ml)	14.5 [12.0–28.5]	13.5 [8.8–34.7]	Mann-Whitney U	0.95

**TABLE 4:** Case summary of APT Group animals. +, present; -, absent. Normal intermittent active fetal swallowing movement, breathing movements, gross fetal body movements, and flexure and extension of limbs were assessed at least every 6 h. A clamp of APT circuit was adjusted to control excessive circuit blood flow (ml/min) below 350 ml/kg/min. When more than a 15% decrease in circuit blood flow within a 6 h period was observed, the clamp was released. When circuit blood flow decreased to less than 150 ml/min, intravenous administration of higher-dose nitroglycerin (2–10 μg/kg/min) was used to prevent constriction of umbilical vessels and maintain circuit blood flow. Bleeding was detected by gross appearance or ultrasound. Edema was defined by ultrasound; skin thickness greater than 5 mm as positive (+). Daily urinary output more than daily infusion volume was defined as positive (+), correct samples from case E were not taken. Samples for microbial culture bottles were collected from fetal umbilical artery, synthetic AF in artificial womb and the sterilized AF storage tub at the end of the experimental period.

Case	A	B	C	D	E	F
Body weight (Induction of APT) (g)	705	630	605	625	672	698
Body weight (Conclusion) (g)	1,460	1,230	1,290	1,250	1,230	1,480
Survival time (hours)	296	306	213	174	173	336
Swallowing movement	+	+	(36H-) +	+	+	+
Breathing movement	+	+	(36H-) +	+	+	+
Gross body movements	+	+	(48H-) +	+	+	+
Flexure and extension of limbs	+	+	(48H-) +	+	+	+
Removal of circuit clamp	84H	84H	108H	132H	160H	126H
High-dose nitroglycerin administration	(240H-) +	-	(210H-) +	-	-	(306H-) +
Bleeding	-	Bladder+ (290H-)	retroperitoneum+ (24H)	Bladder + (172H-)	Bladder + (170H-)	-
Edema	(240H-) +	(216H-) +	-	-	-	(240H-) +
Urinary output	+	+	+	+	N/A	+
Blood culture	-	-	-	-	-	-
Culture from synthetic amniotic fluid in artificial uterus	-	-	-	-	-	-

### Urine variables in the APT group

Reliable urine samples could not be collected for the first 96 h from APT Group fetus Case E, and values were not presented for this animal. The urine output of the remaining five APT animals exceeded infusion volume over the experiments. The dose of amino acids administered was higher than protein excretion into the urine during the experiments (Table 5). The mean tubular reabsorption of phosphorus was 96.1 ± 2.8 (%) (Table 3).

**TABLE 5:** Balance of water and protein between fetal infusion and urine output throughout experiments in the APT group. Reliable urine samples for APT, animal case E could not be collected for first 96 h and are not shown. Infusion volume (ml/h) for each APT Group animal was calculated by the formula: sum of infusion throughout experiment (ml)/experimental duration (h). Voiding volume was measured and urine samples were collected every 24 h from urachus catheter. Average voiding volume (ml/h) was calculated by the following formula: sum of voiding volume throughout experiment (ml)/experimental duration (h). Amino acids administration (g/day) were calculated by the following formula: sum of amino acids administration throughout experiment (g)/experimental duration (h) x 24. Protein excretion into urine (g/d) was calculated by the formula; sum of daily voiding volume (ml) x urine protein concentration (g/ml)/experimental duration (h) x 24. Amino acid administration (1 g) was comprised of L-Isoleucine (85 mg), L-Leucine (135 mg), L-Lysine (80 mg), L-Methionine (39 mg), L-Phenylalanine (77 mg), L-Threonine (48 mg), L-Tryptophan (16 mg), L-Valine (90 mg), L-Cysteine (10 mg), L-Tyrosine (5 mg), L-Arginine (111 mg), L-Histidine (47 mg), L-Alanine (86 mg), L-Aspartic acid (5 mg), L-Glutamic acid (5 mg), glycine (55 mg), L-Proline (64 mg), L-Serine (42 mg).

Case	A	B	C	D	E	F
Infusion volume (ml/h)	8.2	8.2	8	8.2	8	6.8
Voiding volume (ml/h)	14.5	14.2	13.7	19.08	—	9.7
Amino acids administration (g/day)	2.31	2.34	2.25	2.25	2.18	2.47
Protein excretion into urine (g/day)	0.022	0.055	0.027	0.035	—	0.032

### Cardiac ultrasound at the end of experiments

Final assessment of cardiac function without overt bleeding detected by macroscopic or ultrasound was used (APT animal case A; 288 h, B; 288 h, C; 210 h, D; 168 h, E; 168 h, F; 336 h). TCD in the APT Group fetuses was significantly shorter than that in the *in-utero* Control Group fetuses. There was no significant difference in tricuspid and mitral valve E/A ratio, MPI, and dimension of the *ductus arteriosus* between the *in-utero* Control Group and the APT Group fetuses. PLI in the APT Group fetuses was significantly higher than that in the *in-utero* Control Group fetuses. The flow direction of *ductus arteriosus* was from right to left in each of the six APT animals (Table 6).

**TABLE 6:** Comparison of fetal cardiac ultrasound data before euthanasia. Eight Control Group animals and Six APT, Group animals were analyzed. Data from the final assessment of cardiac function in the absence of overt bleeding were used as cardiac ultrasound data before euthanasia (APT, animal case A; 288 h, B; 288 h, C; 210 h, D; 168 h, E; 168 h, F; 336 h). Values are expressed as the group mean ± SD. t-tests was conducted for statistical analyses. *P* < 0.05 was considered as significant difference. Blood flow from the pulmonary artery to the descending aorta was determined as right to left directional flow.

	Control group	APT group	*p*-value
Total cardiac dimension (mm)	28.6 ± 3.9	24.6 ± 1.4*	0.024 [−7.3 to −0.6]
Tricuspid valve E/A ratio	0.69 ± 0.07	0.71 ± 0.07	0.591
Mitral valve E/A ratio	0.72 ± 0.07	0.72 ± 0.06	0.912
Myocardial performance index	0.38 ± 0.05	0.40 ± 0.10	0.695
Preload index	0.39 ± 0.07	0.49 ± 0.05 *	0.013 [0.02–0.17]
Dimension of *ductus arteriosus* (mm)	4.5 ± 0.6	4.3 ± 0.9	0.556
Direction of *ductus arteriosus* flow	Right → Left	Right → Left	

### Individual case information

Individual case information (A-F) for each of the six APT Group fetuses is as follows, with data summarized in Figure 1; Table 4:

**Case A):** Normal movements (intermittent active fetal swallowing movements, breathing movements, gross fetal body movements, and flexure and extension of limbs) were observed throughout the assessed period. The circuit clamp was removed at 84 h. Otherwise, all measured physical variables remained within the respective reference ranges from 0 h to 236 h. Circuit blood flow declined sharply at 236 h without overt occlusion of the APT circuit or evidence of bleeding. High-dose nitrogen administration was performed to control intermittent spasmic plunge of circuit blood flow. Although nitrogen administration assisted with circuit blood flow maintenance, circuit blood flow deteriorated after 264 h, and the animal was euthanized at 296 h. Edema was detected at and after 240 h. Daily urinary output constantly exceeded daily infusion volume. No bacteria were identified from blood and AF culture bottles at the experimental conclusion.

**Case B):** Normal movements were observed throughout the assessed period. The circuit clamp was removed at 84 h. Otherwise, all measured physical variables remained within the respective reference ranges from 0h to 288 h. Gross hematuria started at 289 h. Although FFP and RBC were administered and Hb level and urination were maintained within reference ranges, circuit blood flow gradually deteriorated, and the animal was euthanized at 306 h. Necropsy revealed that the urachal catheter had perforated the bladder wall, resulting in bleeding. Edema was detected at and after 216 h. Daily urinary output constantly exceeded daily infusion volume. No bacteria were identified from blood and AF culture bottles at the experimental conclusion.

**Case C):** Fetus had retroperitoneal bleeding at 24 h, resulting in reduced circuit blood flow and elevated lactate levels. RBC (20 ml/kg), FFP (10 ml/kg) and midazolam (0.2 mg/kg) were administered. Measured variables recovered by 26 h and remained within their respective reference ranges from 26 h to 236 h apart from the removal of the circuit clamp at 108 h. Then, normal movements were observed constantly after 36 h. Although high-dose nitrogen administration was commenced to control intermittent spasmic plunge of circuit blood flow at 210 h, circuit blood flow further deteriorated, and the fetus was euthanized at 213 h. Edema was not detected. Daily urinary output constantly exceeded daily infusion volume. No bacteria were identified from blood and AF culture bottles at the conclusion of the experiment.

**Case D):** Normal movements were observed throughout the assessed period. The circuit clamp was removed at 132 h. Otherwise, all measured physical variables remained within the respective reference ranges from 0h to 173 h. Gross hematuria started at 173 h. Although FFP and RBC were administered, circuit blood flow sharply deteriorated and the animal was euthanized at 174 h. Necropsy revealed that the urachal catheter had perforated the bladder and colon, resulting in bleeding. Edema was not detected. Daily urinary output constantly exceeded daily infusion volume. No bacteria were identified from blood and AF culture bottles at the end of the experiment.

**Case E):** Normal movements were observed throughout the assessed period. The circuit clamp was removed at 160 h. Otherwise, all measured physical variables remained within the respective reference ranges from 0 to 168 h. Gross hematuria started at 169 h. Although FFP and RBC were administered, circuit blood flow sharply deteriorated and the fetus was euthanized at 173 h. Necropsy revealed that the urachal catheter had perforated the bladder resulting in bleeding. Edema was not detected at 168 h. Daily urinary output constantly exceeded daily infusion volume. No bacteria were identified from blood and AF culture bottles at the conclusion of the experiment.

**Case F):** Normal movements were observed throughout the assessed period. Circuit blood flow from one artery suddenly declined and suspended without overt occlusion of the APT circuit and bleeding at 126 h. The circuit clamp was removed at this time. High-dose nitrogen administration was performed to control intermittent spasmic plunge of circuit blood flow at 306 h. Otherwise, all measured physical variables remained within the respective reference ranges throughout the experiment. Edema was detected at and after 240 h. Daily urinary output consistently exceeded daily infusion volume. No bacteria were identified from blood and AF culture bottles at the end of the experiment.

## Discussion

### Principal findings

The key findings of this study are:

i) the successful maintenance of key physiologic variables and circulation with pharmacological support for up to 2 weeks in extremely preterm ovine fetuses maintained on an artificial placenta platform without evidence of infection; and ii) a reduction in the growth trajectory of APT-treated fetuses, relative to *in-utero* Control Group animals (Figures 1, 2; Table 1).

To our knowledge, this is the first report of an artificial placenta platform being successfully used to achieve 2 weeks maintenance of extremely preterm fetuses (∼600 g) approximating the size and weight of a human fetus close to the border of viability (21–24 weeks of gestation). This is an important advance, as it means that the field can begin to focus its attention on resolving the complex question of how to achieve normal fetal growth and organ development in APT-treated extremely preterm fetuses–the eventual target population for this therapy.

In the present study, APT fetuses had mostly stable hemodynamic parameters (Figure 1, 2; Tables 2–6). All animals were free of bacteraemia and no systemic inflammatory changes were detected (Tables 3, 4). Three APT Group fetuses had bleeding, predominantly from injury caused by the urachal catheter. The need to place such a catheter in order to drain urine from the bladder, and the resultant risk of injury is likely an ovine-specific challenge (i.e., unlikely required in the human) and, in the sheep, likely resolved with the use of a more sophisticated (i.e., less injurious) catheter design. A further two APT Group fetuses had progressive deterioration of APT circuit flow due, as best as we can determine, to constriction of umbilical arteries (Figures 1, 2; Table 4). APT animals also had slowly progressive edema on and after approximately 240 h, presumably due to hypoalbuminemia induced by inappropriate delivery of minerals and nutrition, and likely insufficient growth factors (Tables 3, 4). It is also important to note that the edema observed in the APT Group animals likely masked reduced somatic growth (as evidenced by reduced organ weights and humerus lengths) relative to *in-utero* Control Group fetuses (Table 1).

### Clinical implications

#### Compatibility of APT platform with extremely preterm fetuses less than 1,000 g

All six animals (100%) scheduled for APT therapy were successfully adapted to the APT platform. Given a reported successful rate of APT transition using mammals that have similar cord anatomy to the human (approximately from 10% to 85%) (Usuda et al., 2019; Charest-Pekeski et al., 2021; Darby et al., 2021), our platform can be successfully used to transition a fetus into APT without technical issues. It is important, however, to note that the fetal sheep, possessing two umbilical veins and two umbilical arteries in a comparably soft and linear umbilical cord, is likely easier to catheterize than that of the extremely preterm human.

Key physiological parameters and blood lactate levels mostly remained within their reference ranges or rapidly returned to reference range (Faber and Green, 1972; Parisi and Walsh, 1989; Assad et al., 2001) after APT was started unless bleeding and/or constriction of umbilical arteries occurred (Figure 1; Table 2). Given previous studies in which incompatibility of the extracorporeal system likely caused progressive circulatory failure in extremely preterm mammals less than 1,000 g, resulting in fetal death (Hornick et al., 2019; Charest-Pekeski et al., 2021; Charest-Pekeski et al., 2022), our APT platform allowed for more stable physiological maintenance, constant fetal (albeit high) urine output and reassuring fetal movement (Tables 2–6). In addition, biochemical tests did not indicate injury of key organs including the liver and kidney (Table 3). Thus, for the first time, the core function of our APT platform appears compatible with the stable, 2 weeks maintenance of extremely preterm fetuses.

#### Fetal infection

Infection is a serious and potentially lethal complication in EPIs, associated with poor growth and adverse long-term neurodevelopmental outcomes (Stoll et al., 2015; Bell et al., 2022; Stoll et al., 2004; Zhao et al., 2017). Although it was uncertain whether EPIs at the border of viability could be maintained without infection on an APT system, this study demonstrated neither bacteremia nor significant differences in hematological data in any of the APT animals (Tables 3, 4). Taking into consideration that rates of infection (approximately 30%–40% of infants <28 weeks GA experience sepsis) in EPIs remain high, this finding could be an important advance (Stoll et al., 2004).

### Research implications and limitations

#### Fetal bleeding

The most common complication of general extracorporeal membranous oxygenator (ECMO) therapy is bleeding, and its incidence varies from 10% to 30% (Sy et al., 2017). Given that the gestational development of fetal brain in sheep is similar to that in humans (McIntosh et al., 1979; Back et al., 2012), detailed studies to assess brain status in APT-treated animals will be important for the future clinical application of APT. In the present study, four APT Group animals had bleeding; one from the retroperitoneum, and three others from the bladder. The observed retroperitoneal bleeding was spontaneous and likely caused by anticoagulation, although nonspontaneous bleeding could be caused by iatrogenic injury during cannulation (Sunga et al., 2012; Kasotakis, 2014). Although a precise APTT range for the extremely preterm sheep fetus is not well known, a standard APTT for EPIs in the human is reported to be 75 ± 28 s, and 1.5 to 2.5 × baseline value for the APTT is generally set as targeted prolongation of the clotting time in running ECMO to prevent thrombosis (Neary et al., 2013; Koster et al., 2019). Accordingly, APTT in the present study was targeted at between 100 and 150 s. However, a standard APTT value for EPIs is originally prolonged compared to that for adults (26–36s) or even term babies (40–60s) (Lippi et al., 2007). Thus, the targeted APTT could be excessive and might increase the risk of bleeding in our APT platform. Given this, only local anticoagulation (circuit coating) could be enough to prevent thrombosis in the APT circuit. Optimizing anticoagulation for EPIs undergoing APT will be needed to carefully balance the risk between bleeding and thrombosis effectively. As catheterization could also be a cause of bleeding, future studies should take note of any similar recurrences during surgical procedures.

In terms of bleeding from the bladder, the most likely cause is injury from the urachal catheter. A urachal catheter was placed to obtain urine as a significant portion of bladder urine flows through the urachus until late in gestation in the ovine fetus (Rita et al., 1998). In addition, factors such as the vertical angle of catheter insertion from the urachus towards the bladder, fetal posture, frequent active fetal movements, and an extended duration of catheter insertion are likely to predispose the fetus to bleeding from the bladder.

#### Abnormal growth

Fetal lung, liver, and kidney weights and humerus length gain in the APT Group were less than those of the *in-utero* Control Group. Ultrasound measurements of TCD showed reduced size in the APT Group relative to the Control Group. Additionally, given the presence of edema in the APT group, body weight was unlikely to accurately reflect fetal growth, despite there being no significant differences in fetal body weight at the end of the experiment between the groups (Tables 1, 4).

Although the precise cause of observed reduced growth is unknown (and likely multifactorial), one cause could be reduced IGF-1 (Table 3). IGF-1 plays a crucial role in fetal development and IGF-1, rather than growth hormone (GH), is the main driver of fetal growth (Hellstrom et al., 2017). It has been reported that EPIs exhibit slower postnatal growth trajectories from birth until 30–32 weeks corrected GA despite increased nutrition. Furthermore, poor perinatal weight gain and low IGF-1 levels have been previously linked (Ehrenkranz et al., 2006; Martin et al., 2009). A lack of IGF-1 may exacerbate many metabolic defects, resulting in abnormal skeletal muscle growth and organ (including brain) development, because the metabolic effects of IGF-1 include stimulation of amino acid and glucose uptake in skeletal muscle, differentiation of preadipocytes, protein synthesis, and reduction of hepatic glucose production (Joseph D'Ercole and Ye, 2008; Hellström et al., 1992; LeRoith and Yakar, 2007). Although the placenta secretes IGF-1 throughout gestation, it is not clear whether placental IGF-1 is secreted into the fetal circulation (Hiden et al., 2009; Baumann et al., 2014). Circulating fetal IGF-1 is mainly derived from the liver (Bona et al., 1994). The AF (which is swallowed and inhaled by the fetus) contains higher IGF-1 concentrations than cord blood during gestation. Thus, this source might be missing in our APT system as well as in current neonatal care for premature infants. Replenishment of IGF-1 may be a future key to increase fetal growth without alteration of nutrition (Jane et al., 2021).

Stable circuit blood flow in any APT system is crucial and depends on fetal blood pressure. Exogenous hydrocortisone was administered in our study to manage severe hypotension refractory to volume expanders and inotropes (Iijima, 2019; Usuda et al., 2019; Usuda et al., 2020). Hydrocortisone dosing was tapered in this study because refractory hypotension is likely to occur for EPIs just after birth or within the first week of life and normalizes by the end of the second week (Iijima, 2019). Nevertheless, plasma cortisol levels were still significantly higher in the APT Group while there was no significant difference in ACTH (Table 3). Although it is not clear that excessive cortisol solely influences fetal growth (Zozaya et al., 2019), it has been reported that the use of hydrocortisone in neonates is associated with growth abnormalities and interferes with the GH–IGF-1 axis at the hypothalamic, pituitary, and target organ levels (Hochberg, 2002; Tijsseling et al., 2018). Thus, optimizing the use of glucocorticoids may also be an important consideration in future studies.

Metabolic Bone Disease (MBD) of prematurity is a disorder of bone health generally featured by hypophosphatemia, hyperphosphatasemia, and late onset of radiological findings of bone demineralization. MBD is frequently observed in newborns <28 weeks of gestation, occurring in 16%–40% of EPIs (Rustico et al., 2014; Ukarapong et al., 2017). In this study, in addition to the discrepancy in humerus length gain between groups, plasma Ca, P, Mg, and ALP levels in the APT Group were lower than those in the *in-utero* Control Group, while PTH was higher in the APT group (Tables 1, 3). Tubular phosphorus reabsorption is above 95% (Table 3). It is reported that hypophosphatemia is the earliest marker of disrupted mineral metabolism, that the normal range of tubular phosphorus reabsorption is 78%–91%, and a value above 95% is a significant marker of insufficient phosphate supplementation in human premature infants (Catache and Leone, 2003; Pohlandt and Mihatsch, 2004; Rustico et al., 2014).

A low serum Ca level is not a reliable screening test and is likely to be affected by hypoalbuminemia (Faienza et al., 2019). PTH was higher in the APT group but within a clinically acceptable range (Catache and Leone, 2003; Faienza et al., 2019). Although elevation of ALP is reportedly suggestive of impaired bone homeostasis despite the absence of clinical signs, ALP levels in the APT Group were not increased (Hung et al., 2011). ALP values could be masked by other mineral deficiencies represented by magnesium and zinc (Ray et al., 2017). Thus, mineral deficiency represented by P in APT animals could be a marker of underlying impaired bone growth. Presently, there is scant knowledge to inform the optimal nutrition mix for extremely preterm sheep fetuses. This, coupled with a lack of tools to accurately predict fetal ovine weights (i.e., a formula to predict fetal body weight using ultrasound measurements, similar to those available for humans) are animal-specific limitations in the present report. Accordingly, further work to optimize fetal nutrition and predict fetal weights may inform improved post-natal growth management of both experimental systems and the human EPI.

#### Edema

Edema was observed in the APT group fetuses and became apparent around 240 h after the induction of APT. The exchange of fluid between the plasma and the interstitium is determined by the hydrostatic and oncotic pressures in each compartment. The relationship between these parameters has traditionally been described as the permeability of the capillary wall x (Delta hydrostatic pressure - Delta oncotic pressure) by Starling’s law (Taylor, 1981). The overt cause of the increase in capillary permeability, such as ischemic or septic changes (in turn inducing cytokine expression) was not observed before edema emerged in any of APT animals, apart from retroperitoneum bleeding in APT animal case C (Figure 1; Tables 2–4) (Colletti et al., 1990; Ohlsson et al., 1990).

Changes in venous pressure result in parallel alterations in capillary hydrostatic pressure. The venous pressure is increased when the blood volume is expanded, augmenting the volume in the venous system. Given stable low lactate level, constant urinary output in excess of infusion volume, and cardiac ultrasound data (Figure 1; Tables 2, 5, 6), it seems unlikely that progressive dysfunction of cardiac and renal excretion caused circulatory failure and elevation of venous pressure. However, real-time monitoring of central venous pressure would be needed for further evaluation in the future. It is also possible that difficult-to-measure fluid intake by fetal swallowing might contribute to excessive precardiac load, resulting in a predisposition to fetal edema. This could be detected as an elevation of the PLI in cardiac ultrasound (Table 6). Thus, restriction of total water intake for EPIs undergoing APT might be a part of a solution.

Hypoalbuminemia (generally due to albumin loss in the urine or intestinal tract and to decreased hepatic albumin synthesis) contributes to a decrease in oncotic pressure, also resulting in edema formation. Plasma albumin level in APT animals was lower than that in the *in-utero* Control group (Table 3). This progressive hypoalbuminemia could be a primary cause of the slowly emerging edema on and after approximately 240 h (Table 4). Proteinuria observed in this study was not sufficient to meet the criteria of nephrotic syndrome which could contribute to the hypoalbuminemia (Table 5) (Downie et al., 2017). Although an unlikely cause, leakage of albumin from the intestinal tract was not evaluated in this study–a potential limitation. Lastly, there could be problems in the process of protein synthesis which may be related to the reduced fetal growth also observed in this study.

#### Constriction of umbilical vessels

Three fetuses had progressive, eventually fatal deterioration of circuit blood flow and circuit arterial pressure, despite constant urination and Hb levels being maintained within reference ranges by blood transfusions (Figures 1B, D, E, 2; Tables 2, 4, 5). The remaining three also had acute or subacute exacerbation of circuit blood flow disruption with constant urination and without bleeding (Figure 1A, C, E). It is unlikely that acute cardiac failure or progressive hypovolemia reduced circuit blood flow, given acceptable Hb values, constant urination, and normal cardiac ultrasound findings (Tables 2, 5, 6).

Although the mechanism is not clear, presumably constriction of umbilical vessels might be due to changes in the vessels themselves. In terms of the umbilical vein, the tip of the venous catheter was placed inside the umbilical ring, as approximately 21% of fetuses have reportedly been shown to have a permanent or a transitory constriction of the UV at the umbilical ring to at least half its intra-amniotic diameter in the second half of pregnancy.^72^ Given that the circuit clamp was removed in first week of treatment, functional or structural changes might occur in the umbilical cord represented and cause umbilical constriction (Table 4) (Labarrere et al., 1985). In addition, autacoids have been postulated as being of great importance in controlling vessel tone because the umbilical vasculature lacks innervation. The fetal umbilical-placental vessels are sensitive to a wide range of vasoconstrictor autacoids (Boura et al., 1994; Paradis and Zhang, 2013). Sympathetic activation to regulate hemostasis could induce vasoconstriction, resulting in progressive exacerbation of circuit blood flow after bleeding (Preckel and von Känel, 2004). Furthermore, endothelial cell-derived relaxing factors such as prostacyclin and nitric oxide (NO) may be responsible for the maintenance of vascular patency in the fetal extracorporeal circulation (Myatt et al., 1991; Izumi et al., 1995). Endothelial degeneration might be caused by the extended duration of arterial catheter placement, resulting in reduced NO. Thus, the administration of nitroglycerin could be transiently effective (Figure 1; Table 4). Compared to more matured fetuses (>1000 g), even slight constriction may yield more severe adverse effects on the circuit blood flow, given the thinner diameter of vessels and lower cardiac output for EPIs.18 The presence of umbilical cord constriction could also hinder the monitoring of fetal blood pressure using circuit blood pressure (Figure 2C). Thus, further study to elucidate these mechanisms and to devise a solution are likely crucial for the long-time maintenance of EPIs under APT system.

#### Other limitations

The small sample numbers used were an experimental limitation of this study. Although time-matched intrauterine control animals were prepared for each APT animal, the dGA for euthanasia was not unified due to the early euthanasia of five of our six APT animals. Given the deviation of individual growth at each dGA, it is naturally preferable that a larger number of animals were employed for this study.

Furthermore, there are limited data to inform assessments of normal physiological parameters such as heart rate, blood pressure, and placental flow for intrauterine extremely preterm fetal sheep at an extremely early gestational age. To evaluate and refine AP physiological management more comprehensively, additional studies to investigate the normal intrauterine physiological parameters would be of great benefit.

An additional experimental limitation of importance is also the lack of a control group employing ventilated extremely premature lambs. As mentioned above, current neonatal care based on ventilation for EPIs still has severe consequences with survival rate, fetal growth, neurodevelopment, and other life-long complications compared to late preterm and term infants. Thus, ventilated control animals might be appropriate in evaluating the utility of the APT system.

Lastly, the primary focus of this report was physiological longer-term maintenance characteristics. We have not reported detailed assessments of growth factors, key organ development, and injury. Extensive protein profiling and individual organ analyses for the brain, heart, lung, liver, and kidney are detailed and complex - requiring stand-alone studies. Clearly, these evaluations are important and will comprise stand-alone reports in the future.

## Conclusion

We report the first use of APT to successfully maintain extremely preterm ovine fetuses for an extended period, up to 336 h (2 weeks). Our data highlight key challenges (bleeding, growth reductions, hypoalbuminemia, and constriction of umbilical vessels) to be overcome in the development and use of APT for extremely early preterm infants. Bleeding from urachal catheters, and uncertainties regarding optimal fetal nutrients and growth may be animal-specific limitations and warrant further study. Irrespective, stability of the systemic circulation, further optimization of anticoagulation, nutrition, and the endocrine environment to support fetal healthy growth are essential for further development of this technology before clinical translation occurs.

## Data Availability

The raw data supporting the conclusion of this article will be made available by the authors, without undue reservation.

## References

[B1] Acar D. B. Kavuncuoğlu S. Çetinkaya M. Petmezci E. Dursun M. Korkmaz O. (2015). Assessment of the place of tubular reabsorption of phosphorus in the diagnosis of osteopenia of prematurity. Turk Pediatri Ars 50 (1), 45–50. 10.5152/tpa.2015.1478 26078696 PMC4462324

[B2] Assad R. S. Lee F. Y. Hanley F. L. (2001). Placental compliance during fetal extracorporeal circulation. J. Appl. physiology 90 (5), 1882–1886. 10.1152/jappl.2001.90.5.1882 11299282

[B3] Back S. A. Riddle A. Dean J. Hohimer A. R. (2012). The instrumented fetal sheep as a model of cerebral white matter injury in the premature infant. Neurotherapeutics 9 (2), 359–370. 10.1007/s13311-012-0108-y 22399133 PMC3337024

[B4] Baumann M. U. Schneider H. Malek A. Palta V. Surbek D. V. Sager R. (2014). Regulation of human trophoblast GLUT1 glucose transporter by insulin-like growth factor I (IGF-I). PLoS One 9 (8), e106037. 10.1371/journal.pone.0106037 25157747 PMC4144961

[B5] Bell E. F. Hintz S. R. Hansen N. I. Bann C. M. Wyckoff M. H. DeMauro S. B. (2022). Mortality, in-hospital morbidity, care practices, and 2-year outcomes for extremely preterm infants in the US, 2013-2018. Jama 327 (3), 248–263. 10.1001/jama.2021.23580 35040888 PMC8767441

[B6] Bona G. Aquili C. Ravanini P. Gallina M. R. Cigolotti A. C. Zaffaroni M. (1994). Growth hormone, insulin-like growth factor-I and somatostatin in human fetus, newborn, mother plasma and amniotic fluid. Panminerva Med. 36 (1), 5–12. 7916454

[B7] Boullata J. I. Gilbert K. Sacks G. Labossiere R. J. Crill C. Goday P. (2014). A.S.P.E.N. Clinical guidelines: parenteral nutrition ordering, order review, compounding, labeling, and dispensing. JPEN J. Parenter. Enter. Nutr. 38 (3), 334–377. 10.1177/0148607114521833 24531708

[B8] Boura A. L. Walters W. A. Read M. A. Leitch I. M. (1994). Autacoids and control of human placental blood flow. Clin. Exp. Pharmacol. Physiol. 21 (10), 737–748. 10.1111/j.1440-1681.1994.tb02441.x 7867224

[B9] Catache M. Leone C. R. (2003). Role of plasma and urinary calcium and phosphorus measurements in early detection of phosphorus deficiency in very low birthweight infants. Acta Paediatr. 92 (1), 76–80. 10.1111/j.1651-2227.2003.tb00473.x 12650304

[B10] Charest-Pekeski A. J. Cho S. K. S. Aujla T. Sun L. Floh A. A. McVey M. J. (2022). Impact of the addition of a centrifugal pump in a preterm miniature pig model of the artificial placenta. Front. physiology 13, 925772. 10.3389/fphys.2022.925772 PMC9356302 35941934

[B11] Charest-Pekeski A. J. Sheta A. Taniguchi L. McVey M. J. Floh A. Sun L. (2021). Achieving sustained extrauterine life: challenges of an artificial placenta in fetal pigs as a model of the preterm human fetus. Physiol. Rep. 9 (5), e14742. 10.14814/phy2.14742 33650787 PMC7923578

[B12] Colletti L. M. Remick D. G. Burtch G. D. Kunkel S. L. Strieter R. M. Campbell D. A. Jr (1990). Role of tumor necrosis factor-alpha in the pathophysiologic alterations after hepatic ischemia/reperfusion injury in the rat. J. Clin. Invest 85 (6), 1936–1943. 10.1172/JCI114656 2161433 PMC296661

[B13] Comline R. S. Silver M. (1970). Daily changes in foetal and maternal blood of conscious pregnant ewes, with catheters in umbilical and uterine vessels. J. physiology 209 (3), 567–586. 10.1113/jphysiol.1970.sp009180 PMC1395543 5499797

[B14] Darby J. R. T. Berry M. J. Quinn M. Holman S. L. Bradshaw E. L. Jesse S. M. (2021). Haemodynamics and cerebral oxygenation of neonatal piglets in the immediate ex utero period supported by mechanical ventilation or ex utero oxygenator. J. physiology 599 (10), 2751–2761. 10.1113/JP280803 33745149

[B15] Downie M. L. Gallibois C. Parekh R. S. Noone D. G. (2017). Nephrotic syndrome in infants and children: pathophysiology and management. Paediatr. Int. Child Health 37 (4), 248–258. 10.1080/20469047.2017.1374003 28914167

[B16] Ehrenkranz R. A. Dusick A. M. Vohr B. R. Wright L. L. Wrage L. A. Poole W. K. (2006). Growth in the neonatal intensive care unit influences neurodevelopmental and growth outcomes of extremely low birth weight infants. Pediatrics 117 (4), 1253–1261. 10.1542/peds.2005-1368 16585322

[B17] Faienza M. F. D'Amato E. Natale M. P. Grano M. Chiarito M. Brunetti G. (2019). Metabolic bone disease of prematurity: diagnosis and management. Front. Pediatr. 7, 143. 10.3389/fped.2019.00143 31032241 PMC6474071

[B18] Faber J. J. Green T. J. (1972). Foetal placental blood flow in the lamb. J. physiology 223 (2), 375–393. 10.1113/jphysiol.1972.sp009853 PMC1331453 5039279

[B19] Giretti I. Correani A. Antognoli L. Monachesi C. Marchionni P. Biagetti C. (2021). Blood urea in preterm infants on routine parenteral nutrition: A multiple linear regression analysis. Clin. Nutr. 40 (1), 153–156. 10.1016/j.clnu.2020.04.039 32423698

[B20] Glass H. C. Costarino A. T. Stayer S. A. Brett C. M. Cladis F. Davis P. J. (2015). Outcomes for extremely premature infants. Anesth. analgesia 120 (6), 1337–1351. 10.1213/ANE.0000000000000705 PMC4438860 25988638

[B21] Gudmundsson S. (1999). Importance of venous flow assessment for clinical decision-making. Eur. J. Obstet. Gynecol. Reprod. Biol. 84 (2), 173–178. 10.1016/s0301-2115(98)00326-1 10428340

[B22] Hellstrom A. Ley D. Hallberg B. Lofqvist C. Hansen-Pupp I. Ramenghi L. A. (2017). IGF-1 as a drug for preterm infants: A step-wise clinical development. Curr. Pharm. Des. 23 (38), 5964–5970. 10.2174/1381612823666171002114545 28969546 PMC5824464

[B23] Hellström A. Ley D. Hansen-Pupp I. Hallberg B. Löfqvist C. van Marter L. Insulin-like growth factor 1 has multisystem effects on foetal and preterm infant development. Acta Paediatr. (Oslo, Nor. 2016;105(6):576–586. 10.1111/apa.13350 PMC5069563 26833743

[B24] Hidaka N. Sugitani M. Fujita Y. Fukushima K. Tsukimori K. Wake N. (2009). Preload index of the inferior vena cava as a possible predictive marker of hydropic changes in fetuses with Ebstein anomaly. J. Ultrasound Med. 28 (10), 1369–1374. 10.7863/jum.2009.28.10.1369 19778884

[B25] Hiden U. Glitzner E. Hartmann M. Desoye G. (2009). Insulin and the IGF system in the human placenta of normal and diabetic pregnancies. J. Anat. 215 (1), 60–68. 10.1111/j.1469-7580.2008.01035.x 19467150 PMC2714639

[B26] Hochberg Z. Mechanisms of steroid impairment of growth. Hormone Res. Paediatr. 2002;58(Suppl. 1)(Suppl. 1):33–38. 10.1159/000064764 12373012

[B27] Hornick M. A. Mejaddam A. Y. McGovern P. E. Hwang G. Han J. Peranteau W. H. (2019). Technical feasibility of umbilical cannulation in midgestation lambs supported by the EXTra-uterine Environment for Neonatal Development (EXTEND). Artif. Organs 43 (12), 1154–1161. 10.1111/aor.13524 31237960

[B28] Howson C. P. Kinney M. V. McDougall L. Lawn J. E. Born too soon: preterm birth matters. Reprod. health. 2013;10 Suppl. 1(Suppl. 1):S1, 10.1186/1742-4755-10-S1-S1 24625113 PMC3828581

[B29] Hung Y-L. Chen P-C. Jeng S-F. Hsieh C-J. Peng S. S-F. Yen R-F. (2011). Serial measurements of serum alkaline phosphatase for early prediction of osteopaenia in preterm infants. J. Paediatr. Child Health 47 (3), 134–139. 10.1111/j.1440-1754.2010.01901.x 21091586

[B30] Iijima S. (2019). Late-onset glucocorticoid-responsive circulatory collapse in premature infants. Pediatr. Neonatol. 60 (6), 603–610. 10.1016/j.pedneo.2019.09.005 31564521

[B31] Izumi H. Makino Y. Shirakawa K. Garfield R. E. (1995). Role of nitric oxide on vasorelaxation in human umbilical artery. Am. J. obstetrics Gynecol. 172 (5), 1477–1484. 10.1016/0002-9378(95)90481-6 7755057

[B32] Jane S. Sara H. Alicia W. Eileen I. C. Steven C. S. Stephanie R. W. (2021). IGF-1 infusion to fetal sheep increases organ growth but not by stimulating nutrient transfer to the fetus. Am. J. Physiology-Endocrinology Metabolism 320 (3), E527–E538. 10.1152/ajpendo.00453.2020 PMC7988781 33427051

[B33] Joseph D'Ercole A. Ye P. (2008). Expanding the mind: insulin-like growth factor I and brain development. Endocrinology 149 (12), 5958–5962. 10.1210/en.2008-0920 18687773 PMC2613055

[B34] Kanagawa T. Kanzaki T. Chiba Y. (2002). Chronologic change in the PLI value at the fetal inferior vena cava in the Japanese fetus. J. Med. Ultrasound 10 (2), 94–98. 10.1016/s0929-6441(09)60028-x

[B35] Kanzaki T. Chiba Y. (1990). Evaluation of the preload condition of the fetus by inferior vena caval blood flow pattern. Fetal diagnosis Ther. 5 (3-4), 168–174. 10.1159/000263589 2130842

[B36] Kasotakis G. (2014). Retroperitoneal and rectus sheath hematomas. Surg. Clin. North Am. 94 (1), 71–76. 10.1016/j.suc.2013.10.007 24267499

[B37] Koster A. Ljajikj E. Faraoni D. (2019). Traditional and non-traditional anticoagulation management during extracorporeal membrane oxygenation. Ann. Cardiothorac. Surg. 8 (1), 129–136. 10.21037/acs.2018.07.03 30854322 PMC6379198

[B38] Labarrere C. Sebastiani M. Siminovich M. Torassa E. Althabe O. (1985). Absence of wharton's jelly around the umbilical arteries: an unusual cause of perinatal mortality. Placenta 6 (6), 555–559. 10.1016/s0143-4004(85)80010-2 3836403

[B39] LeRoith D. Yakar S. (2007). Mechanisms of disease: metabolic effects of growth hormone and insulin-like growth factor 1. Nat. Clin. Pract. Endocrinol. metabolism 3 (3), 302–310. 10.1038/ncpendmet0427 17315038

[B40] Lippi G. Salvagno G. L. Rugolotto S. Chiaffoni G. P. Padovani E. M. Franchini M. (2007). Routine coagulation tests in newborn and young infants. J. Thromb. Thrombolysis 24 (2), 153–155. 10.1007/s11239-007-0046-4 17510751

[B41] Martin C. R. Brown Y. F. Ehrenkranz R. A. O'Shea T. M. Allred E. N. Belfort M. B. (2009). Nutritional practices and growth velocity in the first month of life in extremely premature infants. Pediatrics 124 (2), 649–657. 10.1542/peds.2008-3258 19651583 PMC2859427

[B42] Mathes M. Maas C. Bleeker C. Vek J. Bernhard W. Peter A. (2018). Effect of increased enteral protein intake on plasma and urinary urea concentrations in preterm infants born at < 32 weeks gestation and < 1500 g birth weight enrolled in a randomized controlled trial - a secondary analysis. BMC Pediatr. 18 (1), 154. 10.1186/s12887-018-1136-5 29739389 PMC5941684

[B43] McIntosh G. H. Baghurst K. I. Potter B. J. Hetzel B. S. (1979). Foetal brain development in the sheep. Neuropathol. Appl. Neurobiol. 5 (2), 103–114. 10.1111/j.1365-2990.1979.tb00664.x 471183

[B44] Miura Y. Matsuda T. Usuda H. Watanabe S. Kitanishi R. Saito M. (2016). A parallelized pumpless artificial placenta system significantly prolonged survival time in a preterm lamb model. Artif. organs 40 (5), E61–E68. 10.1111/aor.12656 26644374

[B45] Miura Y. Saito M. Usuda H. Woodward E. Rittenschober-Böhm J. Kannan P. S. (2015). *Ex-vivo* uterine environment (EVE) therapy induced limited fetal inflammation in a premature lamb model. PLoS One 10 (10), e0140701. 10.1371/journal.pone.0140701 26473607 PMC4608829

[B46] Miura Y. Usuda H. Watanabe S. Woodward E. Saito M. Musk G. C. (2017). Stable control of physiological parameters, but not infection, in preterm lambs maintained on *ex vivo* uterine environment therapy. Artif. organs 41 (10), 959–968. 10.1111/aor.12974 28891072

[B47] Myatt L. Brewer A. Brockman D. E. (1991). The action of nitric oxide in the perfused human fetal-placental circulation. Am. J. obstetrics Gynecol. 164 (2), 687–692. 10.1016/s0002-9378(11)80047-5 1899534

[B48] Neary E. Okafor I. Al-Awaysheh F. Kirkham C. Sheehan K. Mooney C. (2013). Laboratory coagulation parameters in extremely premature infants born earlier than 27 gestational weeks upon admission to a neonatal intensive care unit. Neonatology 104 (3), 222–227. 10.1159/000353366 24030102

[B49] Ohlsson K. Björk P. Bergenfeldt M. Hageman R. Thompson R. C. (1990). Interleukin-1 receptor antagonist reduces mortality from endotoxin shock. Nature 348 (6301), 550–552. 10.1038/348550a0 2147233

[B50] Paradis A. Zhang L. (2013). Role of endothelin in uteroplacental circulation and fetal vascular function. Curr. Vasc. Pharmacol. 11 (5), 594–605. 10.2174/1570161111311050004 24063378 PMC3914771

[B51] Parisi V. M. Walsh S. W. (1989). Fetal vascular responses to prostacyclin. Am. J. obstetrics Gynecol. 160 (4), 871–876. ; discussion 6-8. 10.1016/0002-9378(89)90303-7 2653040

[B52] Partridge E. A. Davey M. G. Hornick M. A. McGovern P. E. Mejaddam A. Y. Vrecenak J. D. (2017). An extra-uterine system to physiologically support the extreme premature lamb. Nat. Commun. 8, 15112. 10.1038/ncomms15112 28440792 PMC5414058

[B53] Pohlandt F. Mihatsch W. A. (2004). Reference values for urinary calcium and phosphorus to prevent osteopenia of prematurity. Pediatr. Nephrol. 19 (11), 1192–1193. 10.1007/s00467-004-1651-5 15349764

[B54] Preckel D. von Känel R. (2004). Regulation of hemostasis by the sympathetic nervous system: any contribution to coronary artery disease? Heartdrug 4 (3), 123–130. 10.1159/000078415 19169370 PMC2629605

[B55] Ramel S. Rao R. (2020). Hyperglycemia in extremely preterm infants. NeoReviews 21 (2), e89–e97. 10.1542/neo.21-2-e89 32005719

[B56] Rasmusen B. A. (1962). Blood groups in sheep. Ann. N. Y. Acad. Sci. 97, 306–319. 10.1111/j.1749-6632.1962.tb34645.x 14490465

[B57] Ray C. S. Singh B. Jena I. Behera S. Ray S. (2017). Low alkaline phosphatase (ALP) in adult population an indicator of zinc (Zn) and magnesium (Mg) deficiency. Curr. Res. Nutr. Food S 5 (3), 347–352. 10.12944/crnfsj.5.3.20

[B58] Rita G. Jeffrey B. Craig A. P. (1998). Premature urachal CLOSURE INDUCES HYDROURETERONEPHROSIS in male fetuses. J. Urology 160 (4), 1463–1467. 10.1016/s0022-5347(01)62592-8 9751394

[B59] Rustico S. E. Calabria A. C. Garber S. J. (2014). Metabolic bone disease of prematurity. J. Clin. Transl. Endocrinol. 1 (3), 85–91. 10.1016/j.jcte.2014.06.004 29159088 PMC5684970

[B60] Snowise S. Johnson A. Copel J. A. D'Alton M. E. Feltovich H. Gratacós E. (2018). 122 - nonimmune hydrops fetalis. Obstetric imaging: Fetal diagnosis and care. Second Edition. China: Elsevier, 519–526.e1.

[B61] Stoll B. J. Hansen N. I. Adams-Chapman I. Fanaroff A. A. Hintz S. R. Vohr B. (2004). Neurodevelopmental and growth impairment among extremely low-birth-weight infants with neonatal infection. Jama 292 (19), 2357–2365. 10.1001/jama.292.19.2357 15547163

[B62] Stoll B. J. Hansen N. I. Bell E. F. Shankaran S. Laptook A. R. Walsh M. C. (2010). Neonatal outcomes of extremely preterm infants from the NICHD Neonatal Research Network. Pediatrics 126 (3), 443–456. 10.1542/peds.2009-2959 20732945 PMC2982806

[B63] Stoll B. J. Hansen N. I. Bell E. F. Walsh M. C. Carlo W. A. Shankaran S. Trends in care practices, morbidity, and mortality of extremely preterm neonates, Jama. 2015;314(10):1039–1051. 10.1001/jama.2015.10244 26348753 PMC4787615

[B64] Sunga K. L. Bellolio M. F. Gilmore R. M. Cabrera D. (2012). Spontaneous retroperitoneal hematoma: etiology, characteristics, management, and outcome. J. Emerg. Med. 43 (2), e157–e161. 10.1016/j.jemermed.2011.06.006 21911282

[B65] Sy E. Sklar M. C. Lequier L. Fan E. Kanji H. D. (2017). Anticoagulation practices and the prevalence of major bleeding, thromboembolic events, and mortality in venoarterial extracorporeal membrane oxygenation: A systematic review and meta-analysis. J. Crit. Care 39, 87–96. 10.1016/j.jcrc.2017.02.014 28237895

[B66] Taylor A. E. (1981). Capillary fluid filtration. Starling forces and lymph flow. Circ. Res. 49 (3), 557–575. 10.1161/01.res.49.3.557 7020975

[B67] Thomas N. (2016). Nutritional care of preterm infants: scientific basis and practical guidelines. Indian J. Med. Res. 143 (4), 531–532. 10.4103/0971-5916.184296

[B68] Tijsseling D. ter Wolbeek M. Derks J. B. de Vries W. B. Heijnen C. J. van Bel F. (2018). Neonatal corticosteroid therapy affects growth patterns in early infancy. PLOS ONE 13 (2), e0192162. 10.1371/journal.pone.0192162 29432424 PMC5809117

[B69] Trends in blood urea nitrogen as a marker of protein intake in extreme preterm infants. J. Paediatr. Child Health. 2017;53(S2):92-.28070951

[B70] Ukarapong S. Venkatarayappa S. K. B. Navarrete C. Berkovitz G. (2017). Risk factors of metabolic bone disease of prematurity. Early Hum. Dev. 112, 29–34. 10.1016/j.earlhumdev.2017.06.010 28683339

[B71] Unno N. Kuwabara Y. Okai T. Kido K. Nakayama H. Kikuchi A. (1993). Development of an artificial placenta: survival of isolated goat fetuses for three weeks with umbilical arteriovenous extracorporeal membrane oxygenation. Artif. Organs 17 (12), 996–1003. 10.1111/j.1525-1594.1993.tb03181.x 8110074

[B72] Usuda H. Watanabe S. Saito M. Ikeda H. Koshinami S. Sato S. (2020). Successful use of an artificial placenta-based life support system to treat extremely preterm ovine fetuses compromised by intrauterine inflammation. Am. J. obstetrics Gynecol. 223 (5), e1–e755. 10.1016/j.ajog.2020.04.036 32380175

[B73] Usuda H. Saito M. Ikeda H. Sato S. Kumagai Y. Saito Y. (2022b). Assessment of synthetic red cell therapy for extremely preterm ovine fetuses maintained on an artificial placenta life-support platform. Artif. Organs 46 (4), 653–665. 10.1111/aor.14155 34932228

[B74] Usuda H. Watanabe S. Miura Y. Saito M. Musk G. C. Rittenschober-Bohm J. (2017). Successful maintenance of key physiological parameters in preterm lambs treated with *ex vivo* uterine environment therapy for a period of 1 week. Am. J. obstetrics Gynecol. 217 (4), e1–e457. 10.1016/j.ajog.2017.05.046 28646647

[B75] Usuda H. Watanabe S. Saito M. Sato S. Musk G. C. Fee M. E. (2019). Successful use of an artificial placenta to support extremely preterm ovine fetuses at the border of viability. Am. J. obstetrics Gynecol. 221 (1), e1–e69. 10.1016/j.ajog.2019.03.001 30853365

[B76] Usuda H. Watanabe S. Saito M. Sato S. Ikeda H. (2022a). Artificial placenta technology: History, potential and perception. China: Placenta. 10.1016/j.placenta.2022.10.003 37743742

[B77] Zeitlin J. Manktelow B. N. Piedvache A. Cuttini M. Boyle E. van Heijst A. (2016). Use of evidence based practices to improve survival without severe morbidity for very preterm infants: results from the EPICE population based cohort. BMJ 354, i2976. 10.1136/bmj.i2976 27381936 PMC4933797

[B78] Zhao X. P. Zhou W. Li X. F. Song Y. Y. Zhang T. Y. Liang H. (2017). Incidence of late-onset sepsis in very low birth weight and extremely low birth weight infants and risk factors for late-onset sepsis. Zhongguo dang dai er ke za zhi = Chin. J. Contemp. Pediatr. 19 (11), 1129–1133. 10.7499/j.issn.1008-8830.2017.11.001 PMC7389318 29132456

[B79] Zozaya C. Avila-Alvarez A. García-Muñoz Rodrigo F. Couce M. L. Arruza L. Fernandez-Perez C. (2019). The impact of postnatal systemic steroids on the growth of preterm infants: A multicenter cohort study. Nutrients 11 (11), 2729. 10.3390/nu11112729 31717933 PMC6893656

